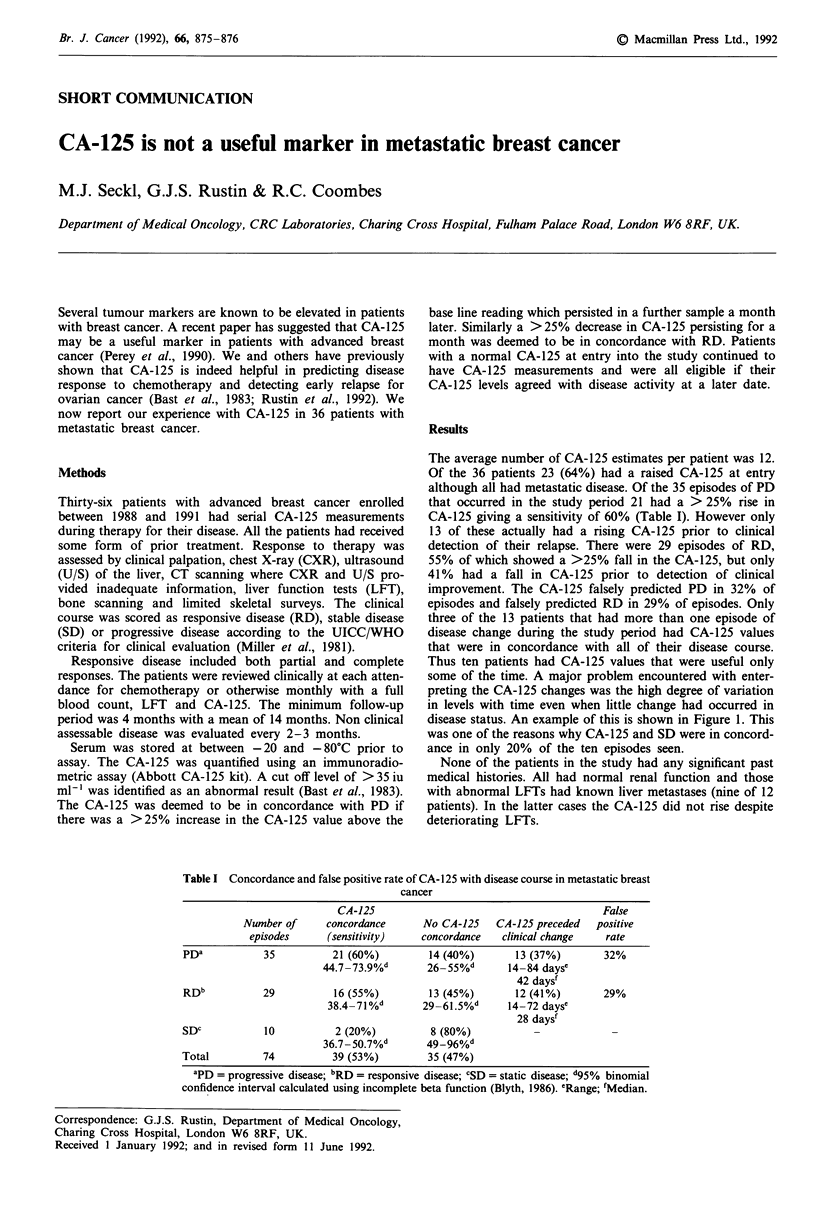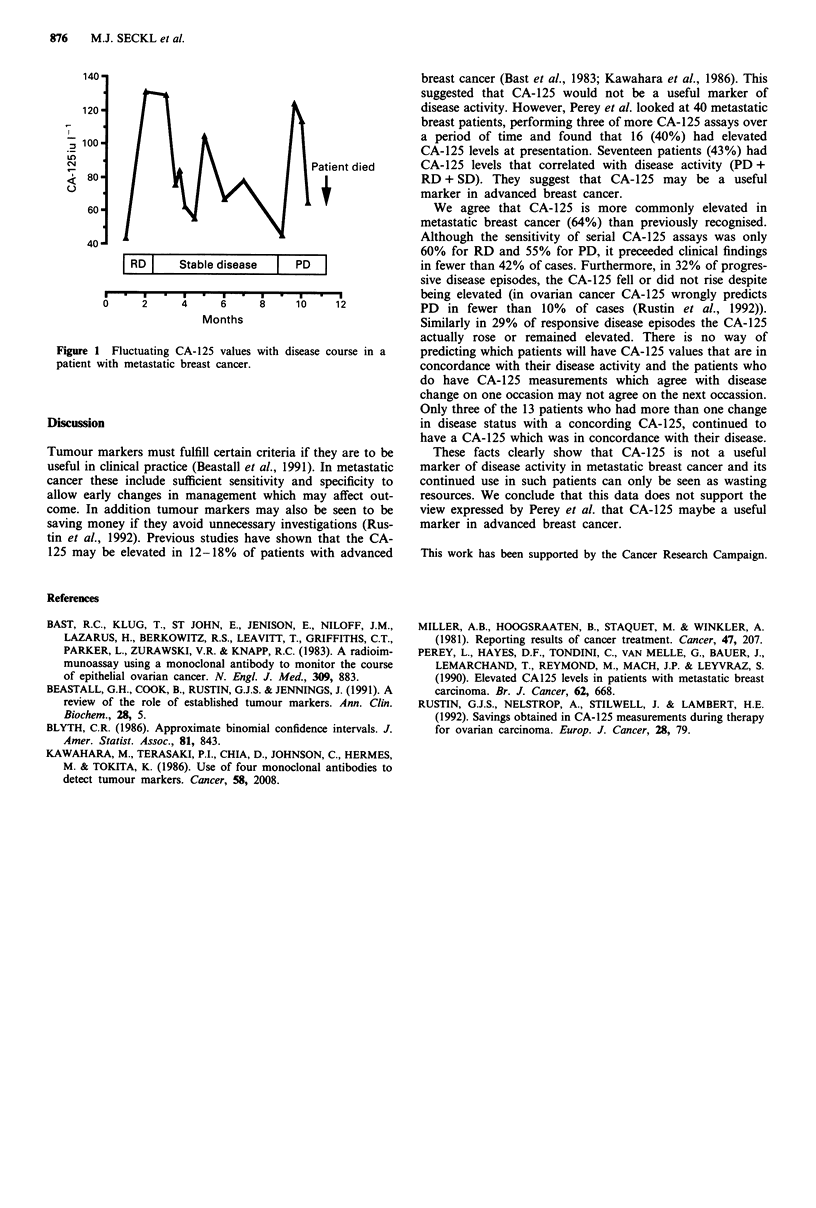# CA-125 is not a useful marker in metastatic breast cancer.

**DOI:** 10.1038/bjc.1992.377

**Published:** 1992-11

**Authors:** M. J. Seckl, G. J. Rustin, R. C. Coombes

**Affiliations:** Department of Medical Oncology, CRC Laboratories, Charing Cross Hospital, London, UK.


Br. J. Cancer (1992), 66, 875–876 © Macmillan Press Ltd., 1992

SHORT COMMUNICATION

### CA-125 is not a useful marker in metastatic breast cancer

M.J. Seckl, G.J.S. Rustin & R.C. Coombes

*Department of Medical Oncology, CRC Laboratories, Charing Cross Hospital, Fulham Palace Road, London W6 8RF, UK.*

Several tumour markers are known to be elevated in patients with breast cancer. A recent paper has suggested that CA-125 may be a useful marker in patients with advanced breast cancer (Perey *et al.*, 1990). We and others have previously shown that CA-125 is indeed helpful in predicting disease response to chemotherapy and detecting early relapse for ovarian cancer (Bast *et al.*, 1983; Rustin *et al.*, 1992). We now report our experience with CA-125 in 36 patients with metastatic breast cancer.

### Methods

Thirty-six patients with advanced breast cancer enrolled between 1988 and 1991 had serial CA-125 measurements during therapy for their disease. All the patients had received some form of prior treatment. Response to therapy was assessed by clinical palpation, chest X-ray (CXR), ultrasound (U/S) of the liver, CT scanning where CXR and U/S provided inadequate information, liver function tests (LFT), bone scanning and limited skeletal surveys. The clinical course was scored as responsive disease (RD), stable disease (SD) or progressive disease according to the UICC/WHO criteria for clinical evaluation (Miller *et al.*, 1981).

Responsive disease included both partial and complete responses. The patients were reviewed clinically at each attendance for chemotherapy or otherwise monthly with a full blood count, LFT and CA-125. The minimum follow-up period was 4 months with a mean of 14 months. Non clinical assessable disease was evaluated every 2–3 months.

Serum was stored at between −20 and −80°C prior to assay. The CA-125 was quantified using an immunoradiometric assay (Abbott CA-125 kit). A cut off level of >35 iu $ml^{-1}$ was identified as an abnormal result (Bast *et al.*, 1983). The CA-125 was deemed to be in concordance with PD if there was a >25% increase in the CA-125 value above the base line reading which persisted in a further sample a month later. Similarly a >25% decrease in CA-125 persisting for a month was deemed to be in concordance with RD. Patients with a normal CA-125 at entry into the study continued to have CA-125 measurements and were all eligible if their CA-125 levels agreed with disease activity at a later date.

### Results

The average number of CA-125 estimates per patient was 12. Of the 36 patients 23 (64%) had a raised CA-125 at entry although all had metastatic disease. Of the 35 episodes of PD that occurred in the study period 21 had a > 25% rise in CA-125 giving a sensitivity of 60% (Table I). However only 13 of these actually had a rising CA-125 prior to clinical detection of their relapse. There were 29 episodes of RD, 55% of which showed a >25% fall in the CA-125, but only 41% had a fall in CA-125 prior to detection of clinical improvement. The CA-125 falsely predicted PD in 32% of episodes and falsely predicted RD in 29% of episodes. Only three of the 13 patients that had more than one episode of disease change during the study period had CA-125 values that were in concordance with all of their disease course. Thus ten patients had CA-125 values that were useful only some of the time. A major problem encountered with enterpreting the CA-125 changes was the high degree of variation in levels with time even when little change had occurred in disease status. An example of this is shown in Figure 1. This was one of the reasons why CA-125 and SD were in concordance in only 20% of the ten episodes seen.

None of the patients in the study had any significant past medical histories. All had normal renal function and those with abnormal LFTs had known liver metastases (nine of 12 patients). In the latter cases the CA-125 did not rise despite deteriorating LFTs.

**Table I** Concordance and false positive rate of CA-125 with disease course in metastatic breast cancer

| | *Number of episodes* | *CA-125 concordance (sensitivity)* | *No CA-125 concordance* | *CA-125 preceded clinical change* | *False positive rate* |
|---|---|---|---|---|---|
| PD[a] | 35 | 21 (60%)<br>44.7–73.9%[d] | 14 (40%)<br>26–55%[d] | 13 (37%)<br>14–84 days[e]<br>42 days[f] | 32% |
| RD[b] | 29 | 16 (55%)<br>38.4–71%[d] | 13 (45%)<br>29–61.5%[d] | 12 (41%)<br>14–72 days[e]<br>28 days[f] | 29% |
| SD[c] | 10 | 2 (20%)<br>36.7–50.7%[d] | 8 (80%)<br>49–96%[d] | – | – |
| Total | 74 | 39 (53%) | 35 (47%) | | |

[a]PD = progressive disease; [b]RD = responsive disease; [c]SD = static disease; [d]95% binomial confidence interval calculated using incomplete beta function (Blyth, 1986). [e]Range; [f]Median.

Correspondence: G.J.S. Rustin, Department of Medical Oncology, Charing Cross Hospital, London W6 8RF, UK.
Received 1 January 1992; and in revised form 11 June 1992.

**Figure 1** Fluctuating CA-125 values with disease course in a patient with metastatic breast cancer.

### Discussion

Tumour markers must fulfill certain criteria if they are to be useful in clinical practice (Beastall *et al.*, 1991). In metastatic cancer these include sufficient sensitivity and specificity to allow early changes in management which may affect outcome. In addition tumour markers may also be seen to be saving money if they avoid unnecessary investigations (Rustin *et al.*, 1992). Previous studies have shown that the CA-125 may be elevated in 12–18% of patients with advanced breast cancer (Bast *et al.*, 1983; Kawahara *et al.*, 1986). This suggested that CA-125 would not be a useful marker of disease activity. However, Perey *et al.* looked at 40 metastatic breast patients, performing three of more CA-125 assays over a period of time and found that 16 (40%) had elevated CA-125 levels at presentation. Seventeen patients (43%) had CA-125 levels that correlated with disease activity (PD + RD + SD). They suggest that CA-125 may be a useful marker in advanced breast cancer.

We agree that CA-125 is more commonly elevated in metastatic breast cancer (64%) than previously recognised. Although the sensitivity of serial CA-125 assays was only 60% for RD and 55% for PD, it preceeded clinical findings in fewer than 42% of cases. Furthermore, in 32% of progressive disease episodes, the CA-125 fell or did not rise despite being elevated (in ovarian cancer CA-125 wrongly predicts PD in fewer than 10% of cases (Rustin *et al.*, 1992)). Similarly in 29% of responsive disease episodes the CA-125 actually rose or remained elevated. There is no way of predicting which patients will have CA-125 values that are in concordance with their disease activity and the patients who do have CA-125 measurements which agree with disease change on one occasion may not agree on the next occassion. Only three of the 13 patients who had more than one change in disease status with a concording CA-125, continued to have a CA-125 which was in concordance with their disease.

These facts clearly show that CA-125 is not a useful marker of disease activity in metastatic breast cancer and its continued use in such patients can only be seen as wasting resources. We conclude that this data does not support the view expressed by Perey *et al.* that CA-125 maybe a useful marker in advanced breast cancer.

This work has been supported by the Cancer Research Campaign.

### References

BAST, R.C., KLUG, T., ST JOHN, E., JENISON, E., NILOFF, J.M., LAZARUS, H., BERKOWITZ, R.S., LEAVITT, T., GRIFFITHS, C.T., PARKER, L., ZURAWSKI, V.R. & KNAPP, R.C. (1983). A radioimmunoassay using a monoclonal antibody to monitor the course of epithelial ovarian cancer. *N. Engl. J. Med.*, **309**, 883.

BEASTALL, G.H., COOK, B., RUSTIN, G.J.S. & JENNINGS, J. (1991). A review of the role of established tumour markers. *Ann. Clin. Biochem.*, **28**, 5.

BLYTH, C.R. (1986). Approximate binomial confidence intervals. *J. Amer. Statist. Assoc.*, **81**, 843.

KAWAHARA, M., TERASAKI, P.I., CHIA, D., JOHNSON, C., HERMES, M. & TOKITA, K. (1986). Use of four monoclonal antibodies to detect tumour markers. *Cancer*, **58**, 2008.

MILLER, A.B., HOOGSRAATEN, B., STAQUET, M. & WINKLER, A. (1981). Reporting results of cancer treatment. *Cancer*, **47**, 207.

PEREY, L., HAYES, D.F., TONDINI, C., VAN MELLE, G., BAUER, J., LEMARCHAND, T., REYMOND, M., MACH, J.P. & LEYVRAZ, S. (1990). Elevated CA125 levels in patients with metastatic breast carcinoma. *Br. J. Cancer*, **62**, 668.

RUSTIN, G.J.S., NELSTROP, A., STILWELL, J. & LAMBERT, H.E. (1992). Savings obtained in CA-125 measurements during therapy for ovarian carcinoma. *Europ. J. Cancer*, **28**, 79.